# Appropriateness of Surgical Antimicrobial Prophylaxis Practices in Australia

**DOI:** 10.1001/jamanetworkopen.2019.15003

**Published:** 2019-11-08

**Authors:** Courtney Ierano, Karin Thursky, Caroline Marshall, Sonia Koning, Rod James, Sandra Johnson, Nabeel Imam, Leon J. Worth, Trisha Peel

**Affiliations:** 1 National Health and Medical Research Council Centre of Research Excellence: National Centre for Antimicrobial Stewardship, Peter Doherty Research Institute for Infection and Immunity, Melbourne, Australia; 2 University of Melbourne, Faculty of Medicine, Dentistry and Health Sciences, Department of Medicine, Royal Melbourne Hospital, Parkville, Australia; 3 Department of Infectious Diseases, Peter MacCallum Cancer Centre, Melbourne, Australia; 4 National Centre for Infections in Cancer, Peter MacCallum Cancer Centre, Melbourne, Australia; 5 Victorian Infectious Diseases Service, Royal Melbourne Hospital, Parkville, Australia; 6 Infection Prevention and Surveillance Service, Royal Melbourne Hospital, Parkville, Australia; 7 Victorian Healthcare Associated Infection Surveillance System, Melbourne, Australia; 8 Sir Peter MacCallum Department of Oncology, University of Melbourne, Melbourne, Australia; 9 Department of Infectious Diseases, Alfred Health/Monash University, Melbourne, Australia

## Abstract

**Importance:**

Surgical antimicrobial prophylaxis (SAP) is a common indication for antibiotic use in hospitals and is associated with high rates of inappropriateness.

**Objective:**

To describe the SAP prescribing practices and assess hospital, surgical, and patient factors associated with appropriate SAP prescribing.

**Design, Setting, and Participants:**

Multicenter, national, quality improvement study with retrospective analysis of data collected from Australian hospitals via Surgical National Antimicrobial Prescribing Survey audits from January 1, 2016, to June 30, 2018. Data were analyzed using multivariable logistic regression. Crude estimates of appropriateness were adjusted for factors included in the model by calculating estimated marginal means and presented as adjusted-appropriateness with 95% confidence intervals.

**Main Outcomes and Measures:**

Adjusted appropriateness and factors associated with inappropriate prescriptions.

**Results:**

A total of 9351 surgical episodes and 15 395 prescriptions (10 740 procedural and 4655 postprocedural) were analyzed. Crude appropriateness of total prescriptions was 48.7% (7492 prescriptions). The adjusted appropriateness of each surgical procedure group was low for procedural SAP, ranging from 33.7% (95% CI, 26.3%-41.2%) for dentoalveolar surgery to 68.9% (95% CI, 63.2%-74.5%) for neurosurgery. The adjusted appropriateness of postprocedural prescriptions was also low, ranging from 21.5% (95% CI, 13.4%-29.7%) for breast surgery to 58.7% (95% CI, 47.9%-69.4%) for ophthalmological procedures. The most common reason for inappropriate procedural SAP was incorrect timing (44.9%), while duration greater than 24 hours was the most common reason for inappropriate postprocedural SAP (54.3%).

**Conclusions and Relevance:**

High rates of inappropriate procedural and postprocedural antimicrobial use were demonstrated across all surgical specialties. Reasons for inappropriateness, such as timing and duration, varied according to the type of SAP and surgical specialty. These findings highlight the need for improvement in SAP prescribing and suggest potential targeted areas for action.

## Key Points

**Question:**

What are the current surgical antimicrobial prophylaxis prescribing practices in Australia, and what factors are associated with their appropriateness?

**Findings:**

This quality improvement study of 9351 surgical episodes found high rates of inappropriate procedural and postprocedural antimicrobial use across various hospital, patient, and surgical factors. The most common reason for inappropriate procedural use was incorrect timing, while duration greater than 24 hours was the most common reason for inappropriate postprocedural use.

**Meaning:**

These findings suggest that the identified hospital, patient, and surgical factors should be considered as targets for development of tailored interventions to ensure appropriateness of surgical antimicrobial prophylaxis prescriptions.

## Teaser

This quality improvement study uses data from the Australian Surgical National Antimicrobial Prescribing Survey to describe prescribing practices for surgical antimicrobial prophylaxis and assess factors associated with appropriate prescribing.

## Introduction

Surgical antimicrobial prophylaxis (SAP) refers to the administration of antimicrobials for the prevention of surgical site infections (SSIs). International data consistently show 12% to 19% of inpatient antimicrobial prescriptions are for SAP.^1,2,3,4,5^ Australian point prevalence data from the Hospital National Antimicrobial Prescribing Survey (Hospital NAPS)^4,5,6,7^ found that 40.3% of surgical prophylaxis prescriptions were classified as inappropriate and 45.2% as noncompliant with Australian national Therapeutic Guidelines.^8,9^ However, point prevalence methods do not capture the complexity of antimicrobial use in surgery, which should be assessed preoperatively and intraoperatively (or procedurally), and post procedurally.

Some procedures (eg, clean procedures such as routine sterile dermatological surgery) do not require SAP.^9,10^ A single dose is recommended when SAP is indicated (eg, clean and clean-contaminated procedures).^9,11,12,13^ Current Australian national guidelines^9^ advocate for single-dose SAP but acknowledge that if postoperative doses are still considered despite lack of quality evidence, then SAP should not continue beyond 24 hours.

Surgical prophylaxis audits have been recommended within the antimicrobial stewardship (AMS) component of the National Safety and Quality Health Service Standards^14,15^ and the national Clinical Care Standard for AMS^16^ in Australia since 2015. In response to the needs of hospital AMS programs, the Surgical NAPS was developed to collect surgery-specific data, including surgery details and timing of antimicrobials, for benchmarking and targeted feedback of SAP prescribing.^17^

This national online auditing platform was implemented to describe SAP prescribing in Australian hospitals. The Surgical NAPS aimed to delineate hospital, surgical, and patient factors associated with appropriate SAP prescribing.

## Methods

### Online Audit Platform Design

The Surgical NAPS online auditing platform has facilitated this multicenter, national, quality improvement study. The platform was codesigned through stakeholder consultation from a range of specialties (ie, infectious diseases, AMS, infection control, anesthesia, surgery) and collaboration with the statewide center responsible for surveillance of SSIs (Victorian Healthcare Associated Infection Surveillance System) and supplemented by a systematic literature review. The Surgical NAPS online platform was built on the existing Hospital NAPS platform and its annual reporting since 2014.^4,5,6,7^ User feedback facilitated survey refinement prior to development and pilot of the web-based survey.

Surgical NAPS data collection and analysis was approved by Melbourne Health Human Research and Ethics Committee. Informed consent was implied when participants agreed to the terms and conditions of the Surgical NAPS online audit platform prior to data submission. This also permitted the use of deidentified data for research purposes. This report was developed in accordance with the Standards for Quality Improvement Reporting Excellence (SQUIRE) reporting guideline.^18^

The Surgical NAPS list of surgical procedures and groups was developed from the Royal Australasian College of Surgeons’ morbidity audit and logbook tool procedure list^19^ and the Fellow of the Royal Australian and New Zealand College of Obstetricians and Gynaecologists’ logbook procedure list and classification list.^20^ When multiple surgical procedure groups were documented for a patient, only the primary procedure system group was included. Definitions of procedural and postprocedural doses are outlined in eTable 1 in the Supplement.

The survey captures patient demographic characteristics (age, sex), clinical information (allergy status, antimicrobial choice, timing, duration) and procedure-related factors (surgical procedure, incision time documentation). Hospital demographics captured include location (state or territory), funding type (public or private), Australian Institute of Health and Welfare peer groups,^21^ and Australian Bureau of Statistics remoteness areas.^22^ The use of Australian Institute of Health and Welfare hospital peer groups categorizes similar hospitals based on shared characteristics and services provided, with a range of categories for both public and private hospitals.

### Data Collection and Collation

The Surgical NAPS audit was conducted annually between January 1, 2016, to June 30, 2018. Survey participation was voluntary and could be completed prospectively or retrospectively. Hospitals could adopt a convenience sampling method to audit either a targeted surgical specialty or all procedures conducted during a specific period.

Trained auditors collected data according to a standardized method and data collection form^17^(eFigure 1 in the Supplement). Auditors were primarily pharmacists, nurses, and infectious disease physicians who were provided with structured education and online training to ensure consistency of methods. All data from registered sites were entered in the Surgical NAPS online audit platform. Ongoing support and advice from a central clinical support team was also available by phone and email to guide auditors, specifically with appropriateness assessments.

Appropriateness was assessed by surgical episode and by each prescription given for the surgery. Three categories of antimicrobial prescriptions were defined for each surgical episode: procedural, postprocedural, and existing (eTable 1 in the Supplement). Guideline compliance was assessed against the national guidelines^8^ or local site-based guidelines available at the time of assessment. A summary of the guideline’s recommendations for the general principles of SAP prescribing is included in eTable 2 in the Supplement. Appropriateness was a composite measure based on antibiotic choice, timing of administration, dose, duration, and repeat dosing applying the standardized Appropriateness Assessment Guide (eFigure 2 in the Supplement). For example, incorrect dosing may refer to a strength that is not consistent with the guidelines or is not clinically appropriate for that specific patient in regard to his or her body weight or renal function.

**Figure 1.**
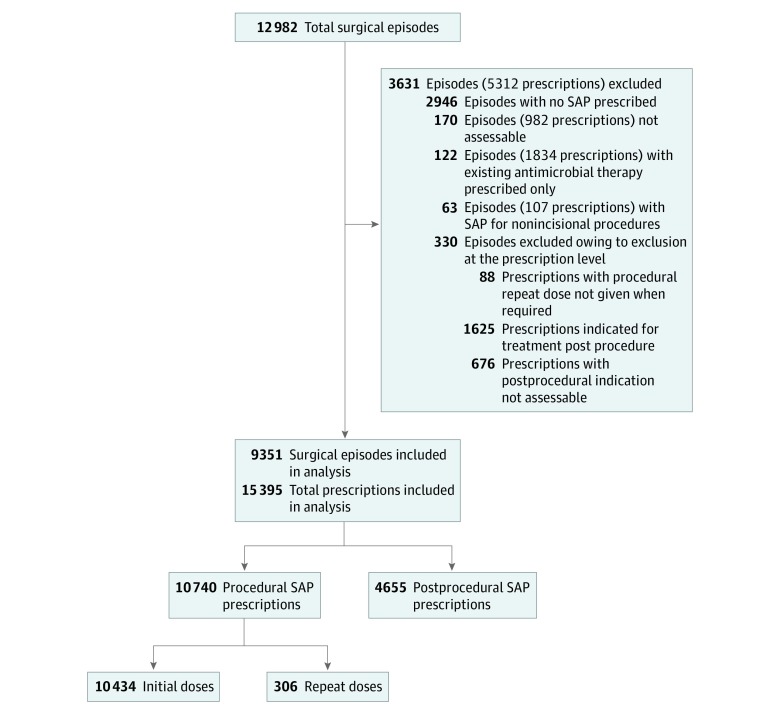
Workflow Summary of Surgical National Antimicrobial Prescribing Survey Analysis SAP indicates surgical antimicrobial prophylaxis.

The purpose of this study was to assess the quality of SAP prescriptions in terms of appropriateness. Surgical episodes and prescriptions in which no antimicrobials were prescribed, existing antimicrobial therapies only, or prescriptions deemed not assessable were therefore excluded from the statistical analysis. Our statistical analysis also excluded repeat doses that were indicated but not given and postprocedural prescriptions with a treatment indication or an indication that was not assessable (Figure 1).

As a sensitivity analysis, crude overall appropriateness of the surgical episode was calculated and compared when antimicrobials were and were not prescribed for either procedural or postprocedural SAP.

For univariable and multivariable analysis, exclusions were doses deemed not assessable or subvariables with missing, low, and/or disproportionate numbers. The top 10 prescribed antimicrobials accounted for more than 90% of the data, and the remaining antimicrobials were classified as “other.”

### Statistical Analysis

Data are presented descriptively, with categorical data presented as frequencies and percentages. Antimicrobial doses were stratified into procedural and postprocedural surgical prophylaxis.

Logistic regression models were used to identify hospital, patient, and surgical factors associated with appropriateness. Model fitting was started with the maximal model, including all relevant factors. Model selection was performed using a likelihood ratio test, and model fit assessed by residual plots. Mixed-effects logistic models fit with unique hospital identifiers as random intercepts provided the best fit to the data and were selected.

Crude estimates of appropriateness were adjusted for factors included in the model by calculating estimated marginal means, presented as adjusted appropriateness (AA) with 95% confidence intervals. These means are generated by estimating the outcome (ie, appropriateness) from the model and calculating an equal-weighted average across all subgroups. Here, the aim is to adjust for any biases caused by varying subgroup sizes in our sample.

Two-tailed tests were conducted, and a *P* value of less than .05 was considered statistically significant. Statistical analysis was conducted with Stata statistical software version 14.1 (StataCorp LP).

## Results

Overall, there were 156 contributing hospitals representing 22.5% of Australian hospitals with the capacity to perform elective procedures (256 public and 437 private hospitals).^21,23,24,25^

In total, data on 12 982 surgical episodes were reviewed, including 6872 female patients (52.9%), 6069 male patients (46.8%), and 41 surgical episodes (0.3%) classified as other; median (range) patient age was 56.5 (0-105) years. Figure 1 provides an overview of included and excluded surgical episodes and antimicrobial prescriptions. There were 3631 surgical episodes excluded from statistical analysis. The remaining 9351 surgical episodes were included in statistical analysis (Figure 1).

A combined total of 15 395 prescriptions (10 740 procedural and 4655 postprocedural) were analyzed. Crude appropriateness of total prescriptions was 48.7% (7492 prescriptions). Hospitals located in Victoria (27.4%) and New South Wales (24.8%) were the largest contributors and accounted for more than half the prescription doses reviewed (eTable 3 and eTable 4 in the Supplement). Public hospitals accounted for 57.5% of the data set in comparison with private hospitals (42.5%). Orthopedic (31.9%) and abdominal (15.2%) surgery were the most commonly audited surgical procedure groups.

Our sensitivity analysis demonstrated a high overall appropriateness per surgical episode when no SAP was prescribed (2767 procedures [93.9%]). Comparatively, overall appropriateness was reduced when at least 1 antimicrobial was prescribed for SAP (3727 procedures [39.9%]). Inversely, inappropriateness per surgical episode when no SAP prescriptions occurred was low (3.9%), compared with 54.1% when an episode included at least 1 prescription for SAP.

### Procedural Prescriptions

**Figure 2.**
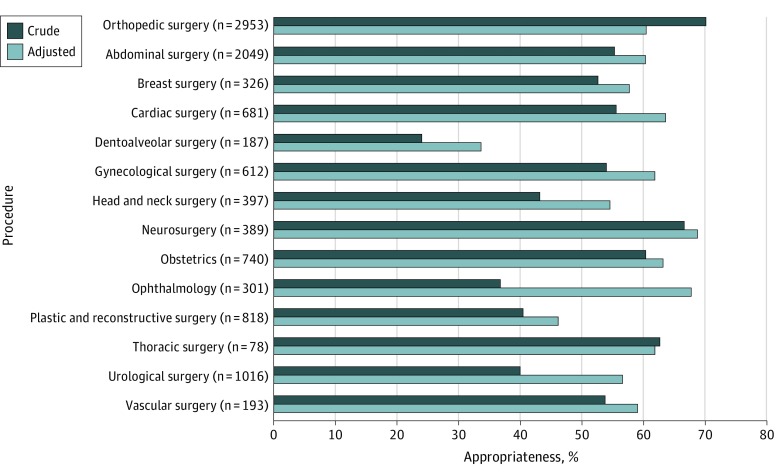
Appropriateness of 10 740 Procedural Prescriptions per Surgical Procedure Groups

Of all 10 740 procedural prescriptions, 615 (5.7%) were not assessable for appropriateness. eTable 3 in the Supplement describes the crude and adjusted appropriateness values for all the variables studied. All factors were included in the multivariable model. Figure 2 illustrates the crude and adjusted appropriateness for each surgical procedure group. The AA of each surgical procedure group was low, ranging from 33.7% (95% CI, 26.3%-41.2%) with dentoalveolar surgery to 68.9% (95% CI, 63.2%-74.5%) for neurosurgery. Similarly, the range of AA across antimicrobial prescriptions was also low (eTable 3 in the Supplement). Ceftriaxone prescriptions accounted for the lowest AA (13.3% [95% CI, 8.4%-18.2%]; 261 prescriptions) and cefoxitin prescriptions for the highest AA (68.3% [95% CI, 58.0%-78.5%]; 77 prescriptions). Ceftriaxone is not recommended at all by national guidelines and cefoxitin is considered a second-line alternative recommendation for specific abdominal procedures.^8^ Cefazolin, the most commonly recommended antimicrobial agent for SAP, had an AA of 64.7% (95% CI, 61.1%-68.3%; 7991 prescriptions) (eTable 3 in the Supplement).

Patient factors such as sex (AA range: 59.1%-59.2%) and age (AA range: 55.0%-61.4%) demonstrated similar AA rates across their respective subvariables. This was similar for surgical factors such as initial vs subsequent admission (AA range: 59.0%-63.0%), surgery classification (AA range: 58.7%-69.8%), and presence of trauma (AA range: 58.7%-66.0%), prosthesis (AA range: 55.7%-65.4%), or excessive blood loss (AA range: 59.0%-65.9%) (eTable 3 in the Supplement).

Subanalysis compared acute hospital peer groups based on their funding type (ie, public or private groups A, B, and C). Significant differences in appropriateness were identified in only 1 comparison, between public acute group B and private acute group B (χ^2^ = 4.03; *P* = .04).

### Postprocedural Prescriptions

A total of 4523 postprocedural surgical prophylaxis prescriptions (97.2%) were included for statistical analysis (132 prescriptions [2.8%] were not assessable). eTable 4 in the Supplement shows the crude and adjusted appropriateness values for all the hospital, patient, and surgical variables that were included in the multivariable model.

After adjustment for these risk factors, AA was low across the hospital variables of state or territory (range: 30.4%-54.6%) and remoteness (range: 42.2%-52.5%) and the patient variables of sex (range: 42.5%-43.6%) and age (range: 40.2%-46.7%) (eTable 4 in the Supplement).

**Figure 3.**
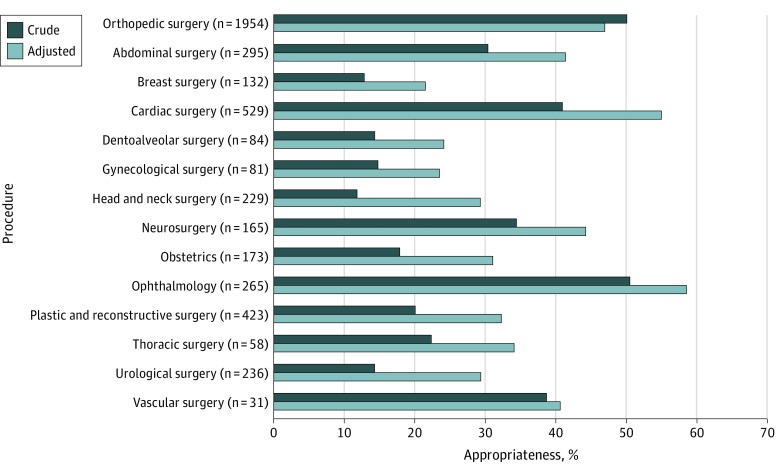
Appropriateness of 4655 Postprocedural Prescriptions per Surgical Procedure Groups

Figure 3 illustrates the crude and adjusted appropriateness for each surgical procedure group. All surgical procedure groups were associated with a low AA, ranging from 21.5% (95% CI, 13.4%-29.7%) for breast surgery to 58.7% (95% CI, 47.9%-69.4%) for ophthalmological procedures. Orthopedic surgery was the most commonly audited procedure group (1954 prescriptions [42.0% of postprocedural prescriptions]) and demonstrated a low AA (47.0% [95% CI, 41.0%-53.1%]). Overall, 38 different antimicrobials were prescribed for postprocedural surgical prophylaxis, with AA ranging from 26.8% for amoxicillin (95% CI, 15.8%-37.7%; 79 prescriptions) to 49.3% for cefazolin (95% CI, 43.6%-55.0%; 2752 prescriptions) (eTable 4 in the Supplement).

Subanalysis of comparable acute hospital peer groups based on their funding type (ie, public or private groups A, B, and C) did not demonstrate significant differences between the respective cohorts (ie, public acute group A vs private acute group A).

### Reasons for Inappropriateness

For procedural prescriptions, 11.7% (1252) were prescribed when procedural SAP was not deemed to be required. For the 9488 procedural prescriptions that were clinically indicated, 33.7% (3195) were inappropriate, with 1 or more reasons for inappropriateness documented (3543 total reasons). A total of 134 of 156 health care facilities (85.9%) contributed to this data subset. Surgical procedure groups with the largest proportion of contributing health care facilities were abdominal (60.4%) and orthopedic (55.2%) procedures. Comparatively, ophthalmology (6.7%) and thoracic (7.5%) surgery had the lowest contribution of different health care facilities.

The reasons for inappropriateness per surgical procedure group are presented in Table 1. The most common reasons for inappropriate prescriptions were incorrect timing (44.9%), incorrect dose (26.1%), and/or spectrum too broad (15.9%). Only 65.6% of surgical episodes included a documented incision time.

**Table 1.** Reasons for Inappropriateness of Procedural Prescriptions per Surgical Procedure Group

Surgical Procedure Groups	No. (%)									
	Health Care Facilities	Inappropriate Prescriptions^a^	Total Reasons for Inappropriateness^b^	Types of Reasons for Inappropriateness						
				Timing^c^	Dose	Broad Spectrum	Narrow Spectrum	Route	Allergy Mismatch	Microbiology Mismatch
Total	134	3195	3534	1588 (44.9)	923 (26.1)	561 (15.9)	279 (7.9)	88 (2.5)	50 (1.4)	45 (1.3)
Orthopedic surgery	74 (55.2)	700 (21.9)	755 (21.4)	360 (47.7)	218 (28.9)	120 (15.9)	31 (4.1)	11 (1.5)	13 (1.7)	2 (0.3)
Abdominal surgery	81 (60.4)	719 (22.5)	789 (22.3)	392 (49.7)	166 (21.0)	116 (14.7)	99 (12.5)	2 (0.3)	8 (1.0)	6 (0.8)
Breast surgery	32 (23.9)	92 (2.9)	79 (2.2)	41 (51.9)	23 (29.1)	3 (3.8)	1 (1.3)	7 (8.9)	3 (3.8)	1 (1.3)
Cardiac surgery	21 (15.7)	204 (6.4)	233 (6.6)	113 (48.5)	42 (18.0)	59 (25.3)	12 (5.2)	ND	3 (1.3)	4 (1.7)
Dentoalveolar surgery	24 (17.9)	78 (2.4)	95 (2.7)	14 (14.7)	28 (29.5)	42 (44.2)	7 (7.4)	ND	1 (1.1)	3 (3.2)
Gynecological surgery	43 (32.1)	176 (5.5)	199 (5.6)	101 (50.8)	64 (32.2)	16 (8.0)	13 (6.5)	1 (0.5)	2 (1.0)	2 (1.0)
Head and Neck surgery	45 (33.6)	97 (3.0)	98 (2.8)	37 (37.8)	32 (32.7)	3 (3.1)	22 (22.4)	1 (1.0)	3 (3.1)	ND
Neurosurgery	31 (23.1)	96 (3.0)	107 (3.0)	47 (43.9)	35(32.7)	9 (8.4)	11 (10.3)	3 (2.8)	2 (1.9)	ND
Obstetrics	42 (31.3)	215 (6.7)	243 (6.9)	153 (63.0)	54 (22.2)	9 (3.7)	11 (4.5)	1 (0.4)	9 (3.7)	6 (2.5)
Ophthalmology	9 (6.7)	60 (1.9)	77 (2.2)	6 (7.8)	23 (29.9)	25 (32.5)	ND	22 (28.6)	1 (1.3)	ND
Plastic and reconstructive surgery	62 (46.3)	226 (7.1)	247 (7.0)	111 (44.9)	83 (33.6)	16 (6.5)	22 (8.9)	5 (2.0)	4 (1.6)	6 (2.4)
Thoracic surgery	10 (7.5)	17 (0.5)	18 (0.5)	11 (61.1)	6 (33.3)	ND	ND	1 (5.6)	ND	ND
Urological surgery	70 (52.2)	457 (14.3)	528 (14.9)	170 (32.2)	124 (23.5)	140 (26.5)	47 (8.9)	34 (6.4)	1 (0.2)	12 (2.3)
Vascular surgery	19 (14.2)	58 (1.8)	66 (1.9)	32 (48.5)	25 (37.9)	3 (4.5)	3 (4.5)	ND	ND	3 (4.5)

Abbreviation: ND, no data.

^a^ Inappropriate prescriptions when surgical antimicrobial prophylaxis was indicated.

^b^ Rationale for the reasons for inappropriateness are described in eFigure 2 in the Supplement: Surgical National Antimicrobial Prescribing Survey Appropriateness Assessment Guide.

^c^ Timing was only assessable for procedural prescriptions.

Of all 4655 postprocedural surgical prophylaxis prescriptions, 60.2% (2801) were deemed inappropriate. The most common reason for inappropriateness was SAP administered when not required (as per guidelines) (61.5% [1724]).

For the remaining 1077 postprocedural prescriptions (38.5%), multiple reasons for inappropriateness could be applied to each prescription (1312 total reasons).

Ninety-eight health care facilities contributed to this data subset. Surgical procedure groups with the largest proportion of contributing health care facilities were orthopedic (66.4%) and plastic and reconstructive (26.5%) procedures. Comparatively, dentoalveolar (2.0%) and ophthalmology (6.1%) surgery had the lowest contribution of different health care facilities.

The reasons for inappropriateness per surgical procedure group are presented in Table 2. Incorrect duration (greater than 24 hours) (54.3%), incorrect dose and/or frequency (28.6%), and spectrum (too broad) (7.5%) accounted for more than 90% of all reasons (Table 2).

**Table 2.** Reasons for Inappropriateness of Postprocedural Prescriptions per Surgical Procedure Group

Surgical Procedure Groups	No. (%)									
	Health Care Facilities	Inappropriate Prescriptions^a^	Total Reasons for Inappropriateness^b^	Types of Reasons for Inappropriateness						
				Incorrect Duration^c^	Dose or Frequency^c^	Broad Spectrum	Narrow Spectrum	Route	Allergy Mismatch	Microbiology Mismatch
Total	98	1077	1312	712 (54.3)	375 (28.6)	99 (7.5)	48 (3.7)	47 (3.6)	10 (0.8)	21 (1.6)
Orthopedic surgery	65 (66.4)	423 (39.3)	499 (38.0)	273 (54.7)	192 (38.5)	16 (3.2)	8 (1.6)	3 (0.6)	6 (1.2)	1 (0.2)
Abdominal surgery	25 (25.5)	52 (4.8)	55 (4.2)	34 (61.8)	6 (10.9)	7 (12.7)	8 (14.5)	ND	ND	ND
Breast surgery	12 (12.2)	48 (4.5)	51 (3.9)	44 (86.3)	7 (13.7)	ND	ND	ND	ND	ND
Cardiac surgery	15 (15.3)	247 (22.9)	399 (30.4)	206 (51.6)	89 (22.3)	33 (8.3)	23 (5.8)	27 (6.8)	3 (0.8)	18 (4.5)
Dentoalveolar surgery	2 (2.0)	12 (1.1)	11 (0.8)	ND	ND	10 (90.9)	1 (9.1)	ND	ND	ND
Gynecological surgery	8 (8.2)	14 (1.3)	16 (1.2)	9 (56.3)	3 (18.8)	3 (18.8)	ND	1 (6.3)	ND	ND
Head and Neck surgery	17 (17.3)	38 (3.5)	22 (1.7)	10 (45.5)	5 (22.7)	4 (18.2)	ND	3 (13.6)	ND	ND
Neurosurgery	14 (14.3)	55 (5.1)	75 (5.7)	43 (57.3)	29 (38.7)	ND	1 (1.3)	1 (1.3)	1 (1.3)	ND
Obstetrics	10 (10.2)	23(2.1)	25 (1.9)	11 (44.0)	5 (20.0)	ND	2 (8.0)	7 (28.0)	ND	ND
Ophthalmology	6 (6.1)	38 (3.5)	32 (2.4)	14 (43.8)	1 (3.1)	17 (53.1)	ND	ND	ND	ND
Plastic and reconstructive surgery	26 (26.5)	49 (4.5)	43 (3.3)	21 (48.8)	13 (30.2)	3 (7.0)	3 (7.0)	2 (4.7)	ND	1 (2.3)
Thoracic surgery	9 (9.2)	17 (1.6)	22 (1.7)	10 (45.5)	8 (36.4)	1 (4.5)	1 (4.5)	1 (4.5)	ND	1 (4.5)
Urological surgery	19 (19.4)	47 94.4)	44 (3.4)	27 (61.4)	9 (20.5)	5 (11.4)	1 (2.3)	2 (4.5)	ND	ND
Vascular surgery	9 (9.2)	14 (1.3)	18 (1.4)	10 (55.6)	8 (44.4)	ND	ND	ND	ND	ND

Abbreviation: ND, no data.

^a^ Inappropriate prescriptions when surgical antimicrobial prophylaxis was indicated.

^b^ Rationale for the reasons for inappropriateness are described in eFigure 2 in the Supplement: Surgical National Antimicrobial Prescribing Survey Appropriateness Assessment Guide.

^c^ Duration and frequency were only assessable for postprocedural prescriptions.

## Discussion

The Surgical NAPS captures real-world prescribing behaviors for SAP across a broad range of procedures, hospital peer groups, and locations from both public and private hospitals. Our analysis has identified key targets for AMS programs, particularly related to timing and duration of SAP. An important finding of this study is that overall rates of appropriateness were low in the surveyed population. Comparatively, when SAP was not prescribed, high rates of appropriateness were demonstrated. Thus, patients not receiving SAP at all were considered a low priority target for SAP optimization.

Our data reveal that there are some interesting differences in rates of appropriateness across surgical specialties in Australian hospitals. Importantly, we did not demonstrate a significant difference between public and private hospital peer groups.

Inappropriate SAP prescribing has consistently been reported in both Australian^26,27,28,29^ and international^30,31,32,33,34,35,36,37,38,39,40,41^ literature with a broad range; Ou et al^40^ reported a 9.4% overall rate of appropriateness, while Hohmann et al^39^ reported an overall compliance rate of 70.7%. The Surgical NAPS data demonstrated SAP appropriateness rates of 53.6% for procedural prescriptions and 36.9% for postprocedural prescriptions, with 48.7% as the combined overall rate of appropriateness. Definitions of appropriateness, compliance, and concordance vary in the literature; thus, interpretation of comparisons requires caution. Our development and application of appropriateness assessments is, to our knowledge, novel in the current literature as it accounts for circumstances in which guideline-noncompliant prescriptions may in fact be appropriate in relation to the prescription’s context.

### Surgical Specialties

The comparison of surgical specialty SAP prescribing is relatively sparse in the current literature, as many assess guideline compliance^34,37,38,39,40,41,42^ as opposed to appropriateness. However, compliance does not infer appropriateness.

Orthopedic surgery was the most commonly audited surgical procedure group, accounting for 31.9% of all prescriptions. Our findings are consistent with the literature, in which orthopedic surgery has demonstrated low SAP guideline compliance^34,37,39,40^ ranging from 24.1%^37^ to 44.4%.^34^

We have identified that all surgical procedure groups demonstrated low AAs across procedural and postprocedural prescriptions. We believe low AAs may have the potential to adversely affect patient care and outcomes. All surgical procedure groups require ongoing support and AMS interventions tailored to their common reasons for inappropriateness to optimize SAP prescribing.

A Chinese prospective multicenter study^40^ assessed the quality of SAP for 14 525 clean and clean-contaminated elective surgical procedures (orthopedic, vascular, gynecologic, and intestinal) and excluded emergency and contaminated and/or dirty procedures. Quality of SAP prescriptions was measured as adherence to the Chinese national guidelines, similar to our methods. Orthopedic surgery was also used as the reference group and gynecological surgery demonstrated significantly high rates of inappropriateness in comparison (odds ratio, 1.60; 95% CI, 1.37-1.88; *P* < .001).^40^ Comparatively, the Surgical NAPS data included 156 hospital sites and emergency and contaminated or dirty procedures. These observations were similar to our results for postprocedural prescriptions, but not to our results for procedural prescriptions.

### Reasons for Inappropriateness and Potential Impact

#### Timing

Incorrect timing was a common reason for inappropriate procedural prescriptions in our surveyed population and the existing literature.^28,31,33,40,43^ It is well established in the current literature that the timing of SAP is associated with rates of SSI.^44,45,46^ Controversy remains when recommending an optimal time frame. The World Health Organization guidelines advocate for administration within 120 minutes of incision time.^46^ Current Australian guidelines recommend antimicrobials with a short half-life (eg, cefazolin) be administered 60 minutes before incision and those with a longer half-life (eg, vancomycin) be administered 120 minutes before incision.^9^

Assessment of appropriate timing depends on the incision time. The Surgical NAPS identified suboptimal documentation of incision time; thus, caution is required when interpreting findings in relation to timing. The Surgical NAPS online reporting and public report^17^ provides ongoing feedback to auditors and highlights this as an area in need of improvement. Implementation of electronic medical records and theater management systems in Australian hospitals are likely to enhance documentation in terms of legibility, completeness, and consistency.^47,48,49,50^ This may support more accurate assessments of timing in relation to first incision.

#### Duration

The Surgical NAPS is the first national survey for SAP that has demonstrated widespread use of extended antimicrobial durations after surgery in Australian hospitals. We believe this use is underestimated, given that the survey permitted up to 24 hours of antibiotics, and not just a single dose, in keeping with published quality indicators.^16,51^

Prolonged duration of antimicrobial use is associated with increased risk of adverse effects, such as *Clostridioides difficile* (formerly *Clostridium difficile*) infection and contributes to the burden of antimicrobial resistance.^52,53,54^ The observational 4-year cohort study by Harbarth et al^55^ of 2641 patients undergoing cardiac surgery confirmed that compared with short SAP (<48 hours), prolonged SAP (>48 hours) was not associated with a decreased risk of SSI (adjusted odds ratio, 1.2; 95% CI, 0.8-1.6) and was associated with an increased risk of acquired antibiotic resistance (adjusted odds ratio, 1.6; 95% CI, 1.1-2.6).

Most recently, a multicenter national retrospective cohort study^56^ of patients in the US Veterans Affairs health care system captured data from 79 058 patients who underwent a range of surgical procedures (cardiac, orthopedic, colorectal, and vascular). The study^56^ demonstrated that longer durations of prophylaxis did not lead to additional SSI reduction and were associated with increases in acute kidney injury and *C difficile* infection.

Recent national and international guidelines^9,11,12^ advocate for single-dose prophylaxis only. Despite the growing evidence advocating for shorter durations and demonstrating harm associated with extended antimicrobial use, duration was identified as the most common reason for inappropriate postprocedural SAP, which mirrors the literature.^28,30,31,32,34,37,40,57^ Our data also highlighted prolonged SAP durations prescribed across a range of procedures in which there was no evidence to support such use.

Retrospective assessments identified postoperative SAP that extended from the operative theater to the ward and discharge prescriptions. Therefore, SAP interventions should not solely target the operating theater and should include recovery and general wards.

### AMS Interventions

The systematic review by Davey et al^58^ demonstrated strong evidence that interventions targeted at antibiotic use among hospital inpatients were associated with increased antibiotic policy compliance and durations. Eleven of the 159 studies (6.9%) with intervention outcomes targeted SAP. Common interventions targeted reducing excessive use.^59,60,61^ Enabling interventions were found to “increase the means and reduce barriers to enhance capability and opportunity”^58^ and were considered the most effective intervention type compared with restrictions of use by expert approval.^62^ Examples of enabling interventions include audit and feedback,^59,60,61,63,64,65^ educational outreach,^63,66^ and clinical decision support systems.^67,68,69^

Adoption of clinical decision support systems may improve evidence-based antimicrobial use.^69^ We support implementation of clinical decision support systems with a variety of interventions such as feedback^58^ from the Surgical NAPS audits to synergistically improve SAP prescribing, feedback, and subsequent outcomes for patients and the health care system.

### Limitations

This study has some limitations. First, participation in the Surgical NAPS is voluntary; thus, our results may not be generalizable across all hospitals. Second, auditor flexibility to perform convenience sampling introduced respondent bias due to the variation in data collected. A mixed-effects regression model was used to account for the intrahospital correlations; however, the effects of an unbalanced survey design may persist. Third, variability of appropriate assessments is probable, as one auditor’s interpretation may have differed from another. To minimize this, an assessment rubric and support from the central clinical support team were available. After completion of the first Surgical NAPS report in 2016,^17^ developers completed a small validation study and demonstrated a 6.7% disagreement rate when comparing assessments by local auditors with those conducted by the NAPS support team. This was deemed acceptable for this type of self-auditing by nonexperts; however, larger validation studies may be warranted.

In addition, there were limited data on clinical outcomes, as this component of the current survey was not mandatory. We support the introduction of mandatory outcome fields and the need for outcome data to provide greater insight and meaning for surgeons. We believe these data are required to drive change in prescribing behaviors. However, collection of data such as SSIs after admission is complex and would require additional systems and resources.

## Conclusions

The Surgical NAPS data have identified high rates of inappropriateness for procedural and postprocedural SAP. Low and wide variation in appropriateness was noted across hospital and surgical factors, in particular surgical procedure groups. Reasons for inappropriateness varied according to the type of SAP, highlighting the need for targeted AMS interventions to address timing for procedural SAP and duration for postprocedural SAP. The Surgical NAPS data set is unique and extensive and continues to grow each year, with more and more Australian surgical centers participating. Ongoing analysis will continue to provide support and direction for AMS interventions, guideline development, and hospital policy.

## Notes

Supplement. **eTable 1.** Surgical NAPS Terminology and Definitions **eFigure 1.** Surgical NAPS Data Collection Form **eTable 2.** Summary of Australian Therapeutic Guidelines: Antibiotic Version 15 Recommendations **eFigure 2.** Surgical NAPS Appropriateness Assessment Guide **eTable 3.** Hospital, Patient and Surgical Factors and Adjusted Appropriateness of Procedural Prescriptions **eTable 4.** Hospital, Patient and Surgical Factors and Adjusted Appropriateness of Post-procedural Prescriptions
