# Supplementary material for: Appropriateness of Surgical Antimicrobial Prophylaxis Practices in Australia
Source: JAMA Netw Open. 2019 Nov 8;2(11):e1915003. doi: 10.1001/jamanetworkopen.2019.15003 (PMC6902799; doi:10.1001/jamanetworkopen.2019.15003)
Supplement: Supplement. — eTable 1. Surgical NAPS Terminology and Definitions eFigure 1. Surgical NAPS Data Collection Form eTable 2. Summary of Australian Therapeutic Guidelines: Antibiotic Version 15 Recommendations eFigure 2. Surgical NAPS Appropriateness Assessment Guide eTable 3. Hospital, Patient and Surgical Factors and Adjusted Appropriateness of Procedural Prescriptions eTable 4. Hospital, Patient and Surgical Factors and Adjusted Appropriateness of Post-procedural Prescriptions [file jamanetwopen-2-e1915003-s001.pdf]

Supplementary Online Content

Ierano C, Thursky K, Marshall C, et al. Appropriateness of surgical antimicrobial prophylaxis in Australia. *JAMA Netw Open*. 2019;2(11):e1915003. doi:10.1001/jamanetworkopen.2019.15003

eTable1. Surgical NAPS Terminology and Definitions

eFigure 1. Surgical NAPS Data Collection Form

eTable 2. Summary of Australian Therapeutic Guidelines: Antibiotic Version 15 Recommendations

eFigure 2. Surgical NAPS Appropriateness Assessment Guide

eTable 3. Hospital, Patient and Surgical Factors and Adjusted Appropriateness of Procedural Prescriptions

eTable 4. Hospital, Patient and Surgical Factors and Adjusted Appropriateness of Post-procedural Prescriptions

This supplementary material has been provided by the authors to give readers additional information about their work.

© 2019 Ierano C et al. *JAMA Network Open.*

eTable1. Surgical NAPS Terminology and Definitions

**Procedural antimicrobial prophylaxis**	All antimicrobials administered either immediately prior to or during the surgical procedure for the purpose of prophylaxis; each dose/prescription of the antimicrobial administered is recorded and reported individually.
**Post-procedural antimicrobial prophylaxis**	All antimicrobials prescribed following, but directly relating to, the procedure for the purposes of prophylaxis; each prescription course of the antimicrobial is recorded and reported, including any inpatient or discharge scripts.
**Existing antimicrobial therapy**	Any antimicrobial prescribed for treatment or prophylaxis in the 24 hours prior (72 hours if on dialysis) to the procedure; these are not analyzed individually but are able to be taken into account when assessing the appropriateness of whether procedural antimicrobials were given or not given.
**Surgical episodes**	Any individual procedure or set of multiple procedures performed together during the one session and the subsequent post-procedural care associated with the procedure(s).
**Appropriateness**	As per the Surgical NAPS Appropriate Assessment Guide (eFigure 2). Data assessed as 'optimal' and 'adequate'.
**Inappropriateness**	As per the Surgical NAPS Appropriate Assessment Guide (eFigure 2). Data assessed as 'suboptimal' and 'inadequate
**Overall Appropriateness /Inappropriateness**	Assessment per entire surgical episode as opposed to the individual antimicrobial doses/prescriptions. e.g. A surgical episode may be deemed to be overall inappropriate if any part of the prescription (procedural or post-procedural prophylaxis) was deemed inappropriate, including allergy or microbiology mismatch, timing, dose, route, frequency, duration, spectrum too broad, spectrum too narrow or if the procedure did not require any antimicrobials.

© 2019 Ierano C et al. *JAMA Network Open.*

eFigure 1. Surgical NAPS Data Collection Form

SURGICAL NAPS National Antimicrobial Prescribing Survey

GUIDANCE | NCAS

Patient identification Number	Date of birth / age	Gender	Date of admission	Date of discharge	Specialty	Height *cm*	Weight *kg*	eGFR / CrCl *ml/min*
	/ /	M / F / O	/ /	/ /				

Surgical details

Surgery date / / **Surgery this admission** ❒ Initial ❒ subsequent

Procedures ❒ emergency ❒ elective ❒ not assessable

❒ trauma ❒ removal/insertion of prosthetic material ❒ excessive blood loss

Surgeon code

Anaesthetist code

Time of first incision : ❒ not documented ❒ not applicable

If not documented or not applicable; surgery start time *(or estimated)* :

End time *(or estimated)* :

Wound classification

❒ clean ❒ clean-contaminated ❒ contaminated ❒ dirty ❒ unknown ❒ not applicable

ASA score ❒ 1 ❒ 2 ❒ 3 ❒ 4 ❒ 5 ❒ 6 ❒ unknown

Risk factors

❒ none identified

All procedures
- ❒ current smoker
- ❒ diabetes
- ❒ peritoneal or haemodialysis
- ❒ obesity (BMI>30)
- ❒ pregnancy
- ❒ rheumatoid arthritis
- ❒ current malignancy
- ❒ previous radiation therapy
- ❒ immunocompromised
- ❒ systemic corticosteroids
- ❒ other immunosuppressive treatments
- ❒ presence of prostheses
- ❒ MRSA colonisation
- ❒ MDR Gram negative colonisation
- ❒ one or more of:
  - prosthetic cardiac valve
  - previous infective endocarditis
  - congenital heart disease with defects
  - rheumatic heart disease in Aboriginal/Torres Strait Islanders

Transrectal prostatic biopsy
- ❒ quinolone therapy in preceding 3 months
- ❒ recent travel to Asia or Southern Europe in preceding 6 months

Gastroduodenal or oesophageal procedures
- ❒ reduced gastric acidity or motility
- ❒ gastrointestinal bleeding
- ❒ gastric outlet obstruction
- ❒ perforation

Biliary surgery
- ❒ acute cholecystitis
- ❒ obstructive jaundice
- ❒ common bile duct stones
- ❒ non-functioning gallbladder

Allergies and adverse drug reactions to antimicrobials

❒ nil known ❒ not documented ❒ present; *specify drug and nature*

Surgical or clinical notes, microbiology, radiology

Existing antimicrobial therapy

Any antimicrobial for treatment or medical prophylaxis or another condition.
*Prescribed in the **24 hours prior** (72 hours if on dialysis) to the procedure*

❒ none prescribed ❒ not assessable

Antimicrobial	Route	Dose	Date and time of last dose
			/ / :
			/ / :
			/ / :
			/ / :

Doc:SurgicalNAPS-DCFv7.20160413

© 2019 Ierano C et al. *JAMA Network Open.*

Peri-operative doses

Include all antimicrobials commenced for the purpose of prophylaxis.

*Record **each dose** on a separate line, including any repeat doses. Include any documented **topical antimicrobials** (e.g. cement beads, soaks, sponges, irrigations, etc.)*

Antimicrobial	Route	Dose	Documented administration time: Not assessable	Nearest 15 minutes	Exact time	Start time	End time	Was this a repeat dose?	Guideline compliance (1-6)	Allergy mismatch	Microbiology mismatch	Incorrect dose	Incorrect route	Incorrect timing	Spectrum too broad	Spectrum too narrow	Peri-operative antimicrobials not required	Procedure requires antimicrobials	Appropriateness (1-5)
						:	:												
						:	:												
						:	:												
						:	:												
❐ ***Repeat dose required, but not given***									4										4
❐ ***No antimicrobial prescribed***																			

Post-operative antimicrobials

*Record those **only relating to the procedure,** including any inpatient or discharge scripts*

Start date and time*	End date and time*	Antimicrobial	Route	Dose	Freq	Indication: For prophylaxis only	For treatment of infection related to the procedure	Not assessable	Guideline compliance (1-6)	Allergy mismatch	Microbiology mismatch	Incorrect dose / frequency	Incorrect route	Incorrect duration	Spectrum too broad	Spectrum too narrow	Post-operative antimicrobials not required	Procedure requires antimicrobials	Appropriateness (1-5)
/ / :	/ / :																		
/ / :	/ / :																		
/ / :	/ / :																		
/ / :	/ / :																		
/ / :	/ / :																		
**If time unknown, write unknown*		❐ ***None prescribed***																	

30 Day follow up

Surgical site infection ❐ none identified ❐ not assessable

❐ identified; *select one type and list any relevant microbiology*

	Microbiology
❐ superficial<br>❐ deep incisional<br>❐ organ space<br>❐ prosthesis	

Clostridium difficile infection	❐ yes	❐ no	❐ unknown
MDR organism	❐ yes	❐ no	❐ unknown
Unplanned ICU admission	❐ yes	❐ no	❐ unknown
Unplanned hospital readmission	❐ yes	❐ no	❐ unknown
Death	❐ yes	❐ no	❐ unknown
Other morbidity *(if yes, specify)*	❐ yes	❐ no	❐ unknown

Guideline compliance

1. Compliant with Therapeutic Guidelines
2. Compliant with locally endorsed guidelines
3. Directed therapy
4. Non-compliant with guidelines
5. No guidelines available
6. Not assessable

Appropriateness

1. Optimal
2. Adequate
3. Sub-optimal
4. Inadequate
5. Not assessable

Doc:SurgicalNAPS-DCFv7;20160413

© 2019 Ierano C et al. *JAMA Network Open.*

eTable 2. Summary of Australian Therapeutic Guidelines: Antibiotic Version 15 Recommendations

eTable 2 was developed as a resource for those who do not have access to the Australian Therapeutic Guidelines version 15, in which the Surgical NAPS Appropriateness Assessments were in relation too. This serves as a brief summary of the guideline's recommendations in relation to the general principles of SAP prescribing.

Antibiotic Prescribing Principles	Summary of recommendations from the Australian Therapeutic Guidelines: Antibiotic Version 15
**Indication**	Do not use SAP unless there is a clear indication for its use i.e. when there is a significant risk of infection or if post-operative infection would have serious consequences.
**Antimicrobial choice**	The prophylactic antibiotic regimen should be directed against the organism(s) most likely to cause postoperative infection.
	Cefazolin is the preferred drug for the majority of procedures that require prophylaxis.
	Modification may be necessary in relation to patient clinical factors (e.g., pre-existing infections, recent antimicrobial use, potential for colonisation of multidrug-resistant organisms, prolonged hospitalisation., presence of prostheses and antibiotic allergy) and environmental factors (e.g., organisms causing infection within the institution and the patterns of antibiotic susceptibility, the potential selection pressure of antibiotic use).
	Strong recommendation to avoid use of broad-spectrum antibiotics for SAP.
**Dose/ Frequency**	Common dosing for cefazolin is 2 grams.
	Dosage adjustment in obesity may be necessary.
	Vancomycin dosing: 15 to 20mg/kg (actual body weight).
	A repeat intraoperative dose is required if the procedure is prolonged or the drug has a short half-life. The interval between pre- and intra-operative doses should be equal to approximately two half-lives of the drug e.g., cefazolin should be administered every four hours.
**Route**	Usually parenteral, intravenous (IV) or intramuscular (IM) are the first line recommendation.
	In some circumstance rectal or oral route may be appropriate.
	Topical antimicrobials for SAP are not recommended.
**Timing**	Timing of SAP should ensure adequate plasma and tissue concentrations are achieved at the time of surgical incision and for the duration of the procedure.
	The optimal time for pre-operative IV SAP administration is within the 60 minutes before surgical incision. Administration 15 to 30 minutes before surgical incision may be optimal.
	Vancomycin infusions should ideally be started 30 to 120 minutes before surgical incision, due to its long infusion time.
**Duration**	A single dose is sufficient for the significant majority of procedures.
	Post-operative doses of IV antibiotics (up to 24 hours) are only required in defined circumstances (e.g. some cardiac and vascular surgeries and lower limb amputation).
	Prophylaxis (IV and oral) should not extend beyond 24 hours, regardless of surgical procedure.
	Urinary or intravascular catheters or indwelling surgical drains are note a justification for the extension of SAP duration.

© 2019 Ierano C et al. *JAMA Network Open.*

eFigure 2. Surgical NAPS Appropriateness assessment guide

SURGICAL NAPS National Antimicrobial Prescribing Survey

Surgical NAPS appropriateness definitions

<table>
<tr><th></th><th colspan="2">Appropriate</th><th colspan="2">Inappropriate</th><th></th></tr>
<tr><th></th><th>1 - Optimal</th><th>2 - Adequate</th><th>3 - Suboptimal</th><th>4 - Inadequate</th><th>5 - Not assessable</th></tr>
<tr><td>Allergy mismatch</td><td rowspan="11">Where antimicrobials are prescribed exactly according to Therapeutic Guidelines or local guidelines – antimicrobial choice, dose, route, timing and duration; or where there is an appropriate reason for deviation from guidelines

If any reason is selected for incorrect prescribing, the prescription will no longer be optimal.</td><td rowspan="4"></td><td>Mild or non-life threatening allergy mismatch</td><td>Life threatening allergy mismatch</td><td rowspan="12">Where there is insufficient information available or the case is too complex for assessment.</td></tr>
<tr><td>Microbiology mismatch</td><td></td><td>Antimicrobial used is too narrow (where sensitivity results available)</td></tr>
<tr><td>Incorrect dose or frequency</td><td>Dose or frequency too high (with exception of gentamicin)</td><td>Dose or frequency too low

Gentamicin dose too high or too frequent</td></tr>
<tr><td>Incorrect route</td><td>An intravenous antimicrobial has been prescribed when the patient is able to safely take it orally</td><td>The prescribed route does not reach the site of infection or surgery</td></tr>
<tr><td>Incorrect timing</td><td>Repeat dose given too soon (including patients who were already on existing antimicrobial therapy)
taking into consideration patients with renal impairment</td><td>Antimicrobial prophylaxis given less than 15 minutes before surgical incision (with exception of vancomycin)

Vancomycin started less than 30 minutes before surgical incision</td><td>Antimicrobial prophylaxis given greater than 60 minutes before surgical incision (with exception of vancomycin)

Vancomycin started greater than 120 minutes before surgical incision

Repeat dose given too late (including patients already on existing antimicrobial therapy)
taking into account renal impairment</td></tr>
<tr><td>Incorrect duration</td><td rowspan="4"></td><td></td><td>Surgical prophylaxis greater than 24 hours (except where guidelines endorse this)</td></tr>
<tr><td>Spectrum too broad</td><td>Choice of antimicrobial is too broad
Additional antimicrobial added unnecessarily</td><td></td></tr>
<tr><td>Spectrum too narrow</td><td rowspan="5"></td><td>Choice of antimicrobial does not cover likely organisms</td></tr>
<tr><td>Procedure does not require any antimicrobials</td><td>Procedure does not require any antimicrobials, but antimicrobials were still prescribed</td></tr>
<tr><td>Procedure requires antimicrobials</td><td>Patient already on existing antimicrobials where last dose would have provided sufficient prophylaxis for the duration of the procedure</td><td>Procedure requires antimicrobials but no antimicrobials were prescribed
AND
there were no existing antimicrobials</td></tr>
<tr><td>Repeat dose required, but not given</td><td rowspan="2"></td><td>This will automatically be selected for auditors</td></tr>
<tr><td>No antimicrobial prescribed</td><td>No antimicrobial required</td><td>Procedure requires antimicrobials but no antimicrobials were prescribed and there were no pre-existing antimicrobials</td></tr>
</table>

Doc:SNAPS.AD.v2; 20170731

© 2019 Ierano C et al. *JAMA Network Open.*

eTable 3. Hospital, patient and surgical factors and adjusted appropriateness of procedural prescriptions

FACTORS	PROCEDURAL PRESCRIPTIONS				
	TOTAL PRESCRIPTIONS	APPROPRIATE (No.) (%)	INAPPROPRIATE (No.) (%)	NOT ASSESSABLE (No.) (%)	MIXED EFFECTS MODEL: ADJUSTED APPROPRIATENESS (%) [95% CI]
**TOTAL**	**10,740**	**5,770 (53.7)**	**4,355 (40.5)**	**615 (5.7)**	**-**
**HOSPITAL FACTORS**					
**STATE/TERRITORY**					
Australian Capital Territory	28	12 (42.9)	16 (57.1)	-	48.5 [11.6-85.3]
New South Wales	2,586	1,553 (60.1)	837 (32.4)	196 (7.6)	70.9 [65.3-76.5]
Northern Territory	50	36 (72.0)	10 (20.0)	4 (8.0)	Not estimable[d]
Queensland	1,962	805 (41.0)	1,053 (53.7)	104 (5.3)	50.1 [41.8-58.4]
South Australia	1,089	636 (58.4)	402 (36.9)	51 (4.7)	61.4 [49.9-72.8]
Tasmania[a]	63	62 (98.4)	1 (1.6)	-	-
Victoria	3,089	1,628 (52.7)	1,249 (40.4)	212 (6.9)	57.1 [50.1-64.1]
Western Australia	1,873	1,038 (55.4)	787 (42.0)	48 (2.6)	55.0 [45.6-64.4]
**ABS REMOTENESS AREA**					
Major cities	8,528	4,681 (54.9)	3,356 (39.4)	491 (5.8)	60.0 [55.7-64.2]
Inner regional	1,716	851 (49.6)	760 (44.3)	105 (6.1)	54.3[45.7-62.9]
Outer regional	415	217 (52.3)	179 (43.1)	19 (4.6)	63.9 [51.7-76.1]
Remote	80	20 (25.0)	60 (75.0)	-	45.7 [19.6-71.8]
Very remote [a]	1	1 (100.0)	-	-	-
**HOSPITAL FUNDING**					
Public	6,222	3,417(54.9)	2,441 (39.2)	364 (5.9)	-
Private	4,518	,2353 (52.1)	1,914 (42.3)	251 (5.6)	-
**HOSPITAL PEER GROUP**					
Principal referral	1,985	1,224 (61.7)	662 (33.4)	99 (5.0)	61.5 [52.0-71.1]
Public acute group A	2,217	1,141 (51.5)	989 (44.6)	87 (3.9)	56.6 [48.9-64.3]
Public acute group B	786	466 (59.3)	301 (38.3)	19 (2.4)	62.1 [51.4-72.9]
Public acute group C	600	265 (44.2)	308 (51.3)	27 (4.5)	56.9 [44.5-69.3]
Public acute group D	7	3 (42.9)	2 (28.6)	2 (28.6)	Not estimable[d]
Mixed sub- & non-acute	69	27 (39.1)	21 (3.0)	21 (3.0)	Not estimable[d]
Mixed day procedure	235	117 (49.8)	86 (36.6)	32 (13.6)	72.3 [51.9-92.7]
Other acute specialised	337	264 (78.3)	69 (20.5)	4 (1.2)	78.0 [63.5-92.6]
Other day procedure	119	3 (2.5)	8 (6.7)	108 (90.8)	43.9 [5.8-82.1]
Women’s hospital	176	91 (51.7)	67 (38.1)	18 (10.2)	56.5 [30.0-83.0]
Children’s hospital	123	63 (51.2)	59 (48.0)	1 (0.8)	63.2 [37.6-88.8]
Unpeered hospitals	17	14 (82.4)	2 (11.8)	1 (5.9)	Not estimable[d]
Private acute group A	517	230 (44.5)	251 (48.5)	36 (7.0)	53.1 [37.9-68.3]
Private acute group B	1,456	602 (41.3)	780 (53.6)	74 (5.1)	46.4 [35.9-56.9]
Private acute group C	593	387 (65.3)	194 (32.7)	12 (2.0)	64.1 [53.1-75.1]
Private acute group D	1,503	873 (58.1)	556 (37.0)	74 (4.9)	64.8 [55.0-74.5]
**PATIENT FACTORS**					
**GENDER**					
Male	4,962	2,627 (52.9)	2,043 (41.2)	292 (5.9)	59.1 [55.4-62.7]
Female	5,727	3,108 (54.3)	2,308 (40.3)	323 (5.6)	59.2 [55.7-62.8]
Unknown[a]	51	35 (68.6)	16 (31.4)	-	-
**AGE (n=10,733)**[b]					
0-19	539	259 (48.1)	267 (49.5)	13(2.4)	55.0 [49.6-60.4]
20-39	2,349	1,264 (53.8)	976 (41.5)	109 (4.6)	58.1 [54.2-62.0]
40-59	2,701	1,456 (53.9)	1,119 (41.4)	126 (4.7)	58.9 [55.1-62.7]

© 2019 Ierano C et al. *JAMA Network Open.*

60-79	4,170	2,292 (55.0)	1,583 (38.0)	295 (7.1)	61.4 [57.8-65.0]
<80	974	494 (50.7)	408(41.9)	72 (7.4)	55.5 [51.1-60.0]
**SURGICAL FACTORS**					
**SURGERY NUMBER FOR ADMISSION**					
Initial	10,302	5,526 (53.6)	4,193 (40.7)	583 (5.7)	59.0 [55.5-62.5]
Subsequent	438	244 (55.7)	162 (37.0)	32 (7.3)	63.0 [57.8-68.2]
**SURGERY CLASSIFICATION**					
Elective	8,670	4,610 (53.2)	3,527 (40.7)	533 (6.1)	58.7 [55.1-62.2]
Emergency	1,893	1,072 (56.6)	759 (40.1)	63 (3.3)	60.3 [56.3-64.3]
Not Assessable	177	89 (50.3)	69 (39.0)	19 (10.7)	69.8 [62.4-77.2]
**TRAUMA**					
Yes	582	371 (63.7)	197 (33.8)	14 (2.4)	66.0 [61.1-71.0]
No	10,158	5,399 (53.2)	4,158 (40.9)	601 (5.9)	58.7 [55.2-62.2]
**PROSTHESIS REMOVAL/INSERTION**					
Yes	3,850	2,308 (59.9)	1,331 (34.6)	211 (5.5)	65.4 [61.8-69.0]
No	6,890	3,462 (50.2)	3,024 (43.9)	404 (5.9)	55.7 [52.0-59.4]
**EXCESSIVE BLOOD LOSS**					
Yes	150	101 (67.3)	46 (30.7)	3 (2.0)	65.9 [58.5-73.4]
No	10,590	5,669 (53.5)	4,309 (40.7)	612 (5.8)	59.0 [55.6-62.5]
**SURGICAL PROCEDURE GROUP**					
Orthopedic surgery	2,953	1,824 (70.3)	1,013 (34.3)	116 (4.5)	60.5 [56.6-64.5]
Abdominal surgery	2,049	1,137 (55.5)	860 (42.0)	52 (2.5)	60.4 [56.4-64.4]
Breast surgery	326	172 (52.8)	117 (35.9)	37 (11.3)	57.8 [51.8-63.8]
Cardiac surgery	681	379 (55.7)	226 (33.2)	76 (11.2)	63.8 [58.4-69.1]
Dentoalveolar surgery	187	45 (24.1)	140 (74.9)	2 (1.1)	33.7 [26.3-41.2]
Gynecological surgery	612	331 (54.1)	245 (40.0)	36 (5.9)	62.0 [57.0-66.9]
Head and Neck surgery	397	172 (43.3)	205 (51.6)	20 (5.0)	54.7 [49.1-60.4]
Neurosurgery	389	260 (66.8)	104 (26.7)	25 (6.4)	68.9 [63.2-74.5]
Obstetrics	740	448 (60.5)	270 (36.5)	22 (3.0)	63.3 [58.2-68.3]
Ophthalmology	301	111 (36.9)	70 (23.3)	120 (39.9)	68.0 [60.1-75.9]
Plastic and reconstructive surgery	818	331 (40.5)	449 (54.9)	38 (4.6)	47.0 [42.3-51.8]
Thoracic surgery	78	49 (62.8)	20 (25.6)	9 (11.5)	61.9 [50.5-73.3]
Urological surgery	1016	407 (40.1)	554 (54.5)	55 (5.4)	56.7 [52.0-61.5]
Vascular surgery	193	104 (53.9)	82 (42.5)	7 (3.6)	59.2 [52.2-66.1]
**ANTIMICROBIAL**					
1.Cefazolin	7,991	4,839 (60.6)	2,827 (35.4)	325 (4.1)	64.7 [61.1-68.3]
2.Metronidazole	692	378 (54.6)	277 (40.0)	37 (5.3)	64.3 [59.5-69.2]
3.Gentamicin	544	120 (22.1)	331 (60.8)	93 (17.1)	28.6 [23.5-33.7]
4. Vancomycin	295	65 (22.0)	192 (65.1)	38 (12.9)	26.3 [20.5-32.1]
5. Ceftriaxone	267	26 (9.7)	236 (88.4)	5 (1.9)	13.3 [8.4-18.2]
6.Piperacillin-tazobactam	131	64 (48.9)	63 (48.1)	4 (3.1)	49.6 [40.7-58.5]
7. Ciprofloxacin	106	13 (12.3)	87 (82.1)	6 (5.7)	21.8 [12.4-31.2]
8. Chloramphenicol	98	6 (6.1)	33 (33.7)	59 (60.2)	18.1 [3.4-32.9]
9. Clindamycin	93	40 (43.0)	50 (53.8)	3 (3.2)	44.5 [35.0-54.0]
10. Cefoxitin	77	55 (71.4)	21 (27.3)	1 (1.3)	68.3 [58.0-78.5]
11. Other (11-31)[c]	446	164 (36.8)	238 (53.4)	44 (9.9)	48.4 [42.5-54.3]

Footnotes:
[a] Excluded due to low/disproportionate numbers
[b] Age data missing for 7 prescriptions and therefore excluded
[c] Other antimicrobials, outside of the top ten most frequently prescribed
[d] AA Not estimable due to low number of prescriptions

© 2019 Ierano C et al. *JAMA Network Open.*

eTable 4. Hospital, patient and surgical factors and adjusted appropriateness of post-procedural prescriptions

FACTORS	POST-PROCEDURAL PRESCRIPTIONS				
	TOTAL PRESCRIPTIONS	APPROPRIATE (No.) (%)	INAPPROPRIATE (No.) (%)	NOT ASSESSABLE (No.) (%)	MIXED EFFECTS MODEL: ADJUSTED APPROPRIATENESS (%) [95% CI]
**TOTAL**	**4655**	**1722 (36.9)**	**2801 (60.2)**	**132 (2.8)**	**-**
**HOSPITAL FACTORS**					
**STATE/TERRITORY**					
Australian Capital Territory	16	1 (6.3)	15 (93.8)	-	Not estimable[d]
New South Wales	1,232	363 (29.5)	849 (68.9)	20 (1.6)	47.4 [37.4-57.3]
Northern Territory	10	7 (70)	3 (30)	-	Not estimable[d]
Queensland	804	220 (27.4)	564 (70.1)	20 (2.5)	30.4[18.9-41.9]
South Australia	516	298 (57.8)	215 (41.7)	3 (0.6)	54.6 [37.8-71.4]
Tasmania[a]	64	64 (100.0)	-	-	-
Victoria	1,122	323 (28.8)	724 (64.5)	75 (6.7)	34.2 [24.1-44.3]
Western Australia	891	446 (50.1)	431 (48.4)	14 (1.6)	50.3 [35.7-64.9]
**ABS REMOTENESS AREA**					
Major cities	3,830	1,405 (36.7)	2,310(60.3)	115 (3.0)	42.2 [36.1-48.4]
Inner regional	711	286 (40.2)	413(58.1)	12 (1.7)	46.9 [34.4-59.5]
Outer regional	98	27 (27.6)	68 (69.4)	3 (3.1)	52.5 [31.8-73.2]
Remote	14	4 (28.6)	8 (57.1)	2 (14.3)	Not estimable[d]
Very remote [a]	2	-	2 (100.0)	-	-
**HOSPITAL FUNDING**					
Public	2,625	971 (37.0)	1,544 (58.8)	110 (4.2)	-
Private	2,030	751 (37.0)	1,257 (61.9)	22 (1.1)	-
**HOSPITAL PEER GROUP**					
Principal referral	952	366 (38.4)	573 (60.2)	13 (1.4)	39.2 [25.7-52.7]
Public acute group A	1,037	393 (37.9)	629 (60.7)	15 (1.4)	42.0 [30.3-53.7]
Public acute group B	205	49 (23.9)	153 (74.6)	3 (1.5)	37.3 [20.6-54.1]
Public acute group C	144	34 (23.6)	97 (67.4)	13 (9.0)	37.1 [17.3-56.8]
Public acute group D	3	-	2 (66.6)	1 (33.3)	-
Mixed sub- & non-acute [a]	0	-	-	-	-
Mixed day procedure	68	30 (44.1)	38 (55.9)	-	Not estimable[d]
Other acute specialised	91	68 (74.7)	20 (22.0)	3 (3.3)	51.3 [21.0-81.5]
Other day procedure	72	-	15 (20.8)	57 (79.2)	-
Women's hospital	21	11 (52.4)	3 (14.3)	7 (33.3)	Not estimable[d]
Children's hospital	88	32 (36.4)	56 (63.6)	-	53.6 [15.8-91.4]
Unpeered hospitals	93	86 (92.5)	6 (6.5)	1 (1.1)	Not estimable[d]
Private acute group A	250	79 (31.6)	163 (65.2)	8 (3.2)	35.5 [13.8-57.2]
Private acute group B	703	178 (25.3)	522 (74.3)	3 (0.4)	33.0 [19.2-46.8]
Private acute group C	377	134 (35.5)	239 (63.4)	4 (1.1)	42.8 [26.6-59.0]
Private acute group D	551	262 (47.5)	285 (51.7)	4 (0.8)	60.4 [46.4-74.4]
**PATIENT FACTORS**					
**GENDER**					
Male	2,255	882 (39.1)	1,312 (58.2)	61 (2.7)	43.6 [38.1-49.0]
Female	2,366	816 (34.5)	1,479 (62.5)	71 (3.0)	42.5 [37.0-48.0]
Unknown[a]	34	24 (70.6)	10 (29.4)	-	-
**AGE (n4,651)[b]**					
0-19	286	94 (32.3)	186 (65)	6 (2.1)	46.7 [39.3-54.1]
20-39	751	214 (28.5)	516 (68.7)	21 (2.8)	42.8 [36.8-48.7]
40-59	955	343 (35.9)	593(62.1)	19 (2.0)	44.1 [38.4-49.9]
60-79	2,104	869 (41.3)	1,164 (55.3)	71 (3.4)	42.9 [37.4-48.4]

© 2019 Ierano C et al. *JAMA Network Open.*

>80	555	201 (36.2)	339 (61.1)	15 (2.7)	40.2 [34.1-46.2]
**SURGERY NUMBER FOR ADMISSION**					
Initial	4,512	1,690 (37.5)	2,695 (59.7)	127 (2.8)	43.3 [37.9-48.7]
Subsequent	143	32 (22.4)	106 (74.1)	5 (3.5)	36.1 [28.3-43.9]
**SURGERY CLASSIFICATION**					
Elective	3,763	1,379 (36.6)	2,275 (60.5)	109 (2.9)	41.1 [35.7-46.5]
Emergency	847	332 (39.2)	495 (58.4)	20 (2.4)	50.8 [44.8-56.8]
Not Assessable	45	11 (24.4)	31 (68.9)	3 (6.7)	47.7 [32.2-63.2]
**TRAUMA**					
Yes	381	153 (40.2)	221 (58.0)	7 (1.8)	42.5 [35.7-49.2]
No	4,274	1,569 (36.7)	2,580 (60.4)	125 (2.9)	43.1 [37.7-48.5]
**PROSTHESIS REMOVAL/INSERTION**					
Yes	2,307	1,017 (44.1)	1,271 (55.1)	19 (0.8)	44.0 [38.4-49.6]
No	2,348	705 (30.0)	1,530 (65.2)	113 (4.8)	42.0 [36.4-47.5]
**EXCESSIVE BLOOD LOSS**					
Yes	70	39 (55.7)	28 (40.0)	3 (4.3)	52.9 [43.4-62.4]
No	4,585	1,683 (36.7)	2,773 (60.5)	129 (2.8)	42.9 [37.5-48.2]
**SURGICAL PROCEDURE GROUP**					
Orthopedic surgery	1,954	981 (50.2)	958 (49.0)	15 (0.8)	47.0 [41.0-53.1]
Abdominal surgery	295	90 (30.5)	203 (68.8)	2 (0.7)	41.5 [34.0-48.9]
Breast surgery	132	17 (12.9)	115 (87.1)	-	21.5 [13.4-29.7]
Cardiac surgery	529	217 (41.0)	308 (58.2)	4 (0.8)	55.1 [47.5-62.6]
Dentoalveolar surgery	84	12 (14.3)	72 (85.7)	-	24.1 [13.2-34.9]
Gynecological surgery	81	12 (14.8)	67 (77.0)	2 (2.5)	23.6 [13.3-33.8]
Head and Neck surgery	229	27 (11.8)	192 (83.8)	10 (4.4)	29.4 [21.7-37.2]
Neurosurgery	165	57 (34.5)	105 (63.6)	3 (1.8)	44.4 [36.6-52.3]
Obstetrics	173	31 (17.9)	127 (73.4)	15 (8.7)	31.1 [22.2-40.1]
Ophthalmology	265	134 (50.6)	70 (26.4)	61 (23.0)	58.7 [47.9-69.4]
Plastic and reconstructive surgery	423	85 (20.1)	330(78.0)	8 (1.9)	32.3 [25.7-38.9]
Thoracic surgery	58	13 (22.4)	44 (75.9)	1 (1.7)	34.2 [22.8-45.7]
Urological surgery	236	34 (14.4)	191 (80.96)	11 (4.7)	29.4 [21.9-36.8]
Vascular surgery	31	12 (38.7)	19 (61.3)	-	40.7 [28.4-53.0]
**ANTIMICROBIAL**					
1. Cefazolin	2,752	1,331 (48.4)	1,395 (50.7)	26 (0.9)	49.3 [43.6-55.0]
2. Cefalexin	520	72 (13.8)	441 (84.8)	7 (1.3)	29.1 [22.7-35.5]
3. Chloramphenicol	245	68 (27.8)	115 (46.9)	62 (25.3)	38.6 [29.5-47.7]
4. Metronidazole	190	37 (19.5)	147(77.4)	6 (3.2)	35.0 [26.7-43.3]
5.Amoxycillin/ Clavulanic Acid	148	31 (20.9)	110 (74.3)	7 (4.7)	43.1 [34.2-51.9]
6. Mupirocin	105	45 (42.9)	57 (54.3)	3 (2.9)	37.3 [29.1-45.5]
7. Vancomycin	89	23 (25.8)	64 (71.9)	2 (2.2)	28.0 [19.6-36.3]
8. Ceftriaxone	79	14 (17.7)	63* (79.7)	2 (2.5)	32.7 [22.8-46.7]
9. Amoxycillin	79	9 (11.4)	68 (86.1)	2 (2.5)	26.8 [15.8-37.7]
10. Gentamicin	67	10 (14.9)	54 (80.6)	3 (4.5)	35.5 [23.8-47.2]
11. Other (11-38)[c]	381	82 (21.5)	287 (75.3)	12 (3.1)	30.1 [23.6-36.6]

Footnotes:

[a] Excluded from multivariate analysis due to low/disproportionate numbers

[b] Age data missing for 4 prescriptions and therefore excluded

[c] Other antimicrobials, outside of the top ten most frequently prescribed

[d] AA Not estimable due to low number of prescriptions

© 2019 Ierano C et al. *JAMA Network Open.*
